# Development of Equipment for Injection Molding of Polymer Products Filled with Recycled Polymer Waste

**DOI:** 10.3390/polym12112725

**Published:** 2020-11-17

**Authors:** Oleg Synyuk, Janusz Musiał, Borys Zlotenko, Tetiana Kulik

**Affiliations:** 1 Department of Machines and Apparatuses, Electromechanical and Power Systems, Khmelnytskyi National University, Instytuts’ka str., 11, 29016 Khmelnytskyi, Ukraine; 2 Faculty of Mechanical Engineering, University of Science and Technology, Kaliskiego 7 Street, 85-789 Bydgoszcz, Poland; janusz.musial@utp.edu.pl; 3 Department of Computer Engineering and Electromechanics, Kyiv National University of Technologies and Design, Nemyrovycha-Danchenka Street, 2, 01011 Kyiv, Ukraine; zlotenco@ukr.net

**Keywords:** light industry equipment, polymer waste, design, polymer structure, deformation, loading, fracture, molding soles, reinforced soles

## Abstract

Polymer waste of light industry and other industries is processed by chemical recycling and mechanical grinding. Modern equipment for polymer waste processing has the following drawbacks: significant energy consumption and reduced performance properties of recycled polymer. New technological processes and equipment for polymer waste recycling have been developed for the manufacture of light industry polymer products with increased performance characteristics. The manufacturing of such products was made possible by the development of the mathematical model, which describes the movement of a mixture of main polymer material and particles of recycled polymer waste in the process of filling a mold cavity. The model, in contrast to the existing models, allows observing the formation of the polymer product structure containing recycled waste particles. Improvement in the performance characteristics of shoe soles made by the injection molding of a mixture of polyvinylchloride and particles of recycled polyvinylchloride was confirmed by experimental tests of breaking strength and fatigue life. The results of these tests can be used in the design of processing equipment to obtain waste particles of the required shape and size and in the design of molds to provide the required concentration and orientation of waste particles in light industry polymer products.

## 1. Introduction

At present, waste recycling of polymer materials is an important issue not only in terms of the natural environment protection, but also due to the fact that in the absence of polymer materials, plastic waste becomes a powerful material and energy resource [1]. There are a lot of problems connected with the utilization of polymer waste. Polymers have their own specific characteristics, but they cannot be considered as insoluble [2]. Effective utilization is impossible without creating effective methods for processing secondary polymer materials and methods for their modification aimed at improving the quality of raw materials without creating special processing equipment or without developing a range of products to be manufactured from secondary polymer materials [3].

Modern technologies for the recycling of light industry polymeric waste include chemical recycling or mechanical grinding of polymers [4]. However, apart from the advantages, chemical recycling has also drawbacks [5]. Chemical processing of polymer waste makes the macromolecular structure of polymers collapse [6]. The complexity of operations and reagents required to recover a monomer suitable for re-polymerization requires significant financial investments; monomers and useful oligomers can be obtained only from a limited set of polymers [7].

The mechanical recycling of polymer waste does not require expensive and sophisticated equipment and can be implemented at any place of waste accumulation [8]. The main advantage of mechanical recycling is that it does not significantly affect the polymer structure. So, technological processes of polymer waste recycling that use mechanical stresses seem to have a big potential [9].

An analysis of recent scientific research results in the field of polymer waste mechanical processing has shown that today, there is almost no equipment for the mechanical processing of polymer waste to provide recycled waste with an oriented structure, which would make them usable as reinforcing elements in the manufacture of light industry products without additional treatment [10]. This implies a necessity to develop a method for designing equipment for polymer waste processing that will ensure destruction of the polymer materials along their structural formation boundaries, thus maintaining the physical and mechanical characteristics of the primary polymer, providing them with anisotropy and reducing the energy consumption [11].

Recycled polymer waste with retained structure can be used in most light industry processes including injection molding, compression molding, extrusion, and calendaring [12,13,14,15,16]. The most useful technological processes are those that have a wide range of technological parameters, i.e., those that do not require narrowing of the specifications range. Therefore, the recycled polymer material is recommended to use in the production of new light industry products, which are obtained by injection molding or extrusion [17,18]. Other methods of forming polymer products of light industry can be used only in cases where the recycled polymer has properties close to the original polymer [19]. The most promising technology today for the production of polymer products using a significant proportion of recycled polymer waste is injection molding.

Polymer products reinforced with particles of crushed polymer material obtain increased physical and mechanical characteristics [20], because the main stresses that arise in the products under the influence of external loads are endured by the polymer filler, and the greatest rigidity and strength of the products is provided along the orientation of the filler particles.

The proposed equipment for processing polymer waste allows obtaining particles that can be used as filler in the production of new polymer products for light industry with enhanced performance, since the particles of recycled polymer waste have an anisotropy of physical and mechanical properties i.e., they have properties similar to reinforcing fibers.

For the production of polymer products of light industry (shoe parts, suitcases, and other products) with the addition of recycled polymer waste, it is necessary to develop molding equipment that provides for the use of recycled polymer particles as a filler or binder.

For reinforced products, it is possible to determine the dominant factor that affects their performance properties, that is, the orientation of the filler particles [21]. All other parameters, such as changes in pressure and temperature, as well as crystallization, are of secondary importance. Thus, the application of known principles of injection molds design to minimize defects does not provide for the enhanced performance of the products; at the same time, completely different approaches are needed.

Since during the filling of a mold with polymer material, filler particles have a certain orientation, the properties of processed material are anisotropic. The use of fillers results in a locally different distribution of stiffness and shrinkage, depending on the orientation of filler particles in the surface area and the wall thickness of the product.

Understanding the processes that occur during the formation of the structure of material with filler particles will allow designing molding equipment to obtain polymer products of light industry with high performance properties [22]. To do this, first of all, it is necessary to simulate the filling process of a mold cavity with polymer material containing inclusions of recycled waste particles.

## 2. Materials and Methods

Materials used are polymer compositions made with the addition of particles of recycled polymer waste with orientation in the matrix in one or more directions. The choice of orientation, concentration, and size of these particles in the main polymer is determined by the distribution of stresses during the operation of polymer products of light industry. This makes it possible to optimize the structure ensuring the strengthening of the material, which allows obtaining polymer products with minimized material consumption.

The manufacturing of such products requires the development of a mathematical model to describe the movement of the mixture of polymer material and particles of recycled polymer waste in the process of filling the mold cavity. The flow charts for the filling process were obtained with the use of the markers and cells method, which realizes a numerical approach to calculating the dynamics of unstable flows of viscous incompressible medium. This method can be used to calculate the motion problems of viscous polymer melt with the positions of polymer material being indicated by markers. Such markers move together with material on the Euler calculation grid.

A finite difference scheme is employed to solve the continuity equation and the equations of motion of continuous medium [5,23]. In the method of markers and cells, all differential equations and boundary conditions are written in finite differences on a grid of fixed cells covering the flow region of the polymer melt. Time is also divided into a finite number of intervals. A system of markers directly connected to polymer material is applied to the volume of polymer and to its surface.

## 3. Results

### 3.1. Mathematical Model of Polymer Melt Motion in Mold Cavity

The flow of a viscous fluid obeys Newton’s law of linear dependence between stress tensors and velocity tensors, which is described with the accuracy required for our problem by the Navier–Stokes equations [24,25]:

(1) $$\rho \frac{dV}{dt}=\rho g-\nabla P+\mu \cdot \nabla^{2}V$$

where *V* is the velocity vector at a single point of polymer medium, (m/s); *μ* is the dynamic viscosity of polymer medium, (Pa·s); *P* is pressure at a single point of polymer melt, (Pa); and *ρ* is the density of polymer medium, (kg/m^3^).

Pressure *P* is determined taking into account the assumption that at any point of the Newtonian viscous fluid, the pressure is equal to the arithmetic mean of three normal stresses applied to mutually perpendicular planes at a certain point of medium, taken with the inverse sign:

(2) $$-\frac{\left(\sigma_{x}+\sigma_{y}+\sigma_{z}\right)}{3}=P$$

where *σ_x_*, *σ_y_*, *σ_z_* are the normal stresses applied to mutually perpendicular areas at a certain point in the medium.

Since the paper considers a stationary density field ($\frac{\partial \rho }{\partial t}=0$), the continuity equation [24,26,27] takes the form:

(3) ∇(ρV)=0

The assumption about the stationary character of the density field is based on the fact that the polymer medium is almost not compressible; i.e., the intermolecular distances do not change. If the movement of polymer medium in a mold is carried out at a relatively low velocity, we can assume that the density is constant at each point of polymer melt, and this allows us obtaining the incompressibility equation for viscous medium:

(4) ∇V=0

Let us rewrite the equation of motion (1) for a polymer melt in Cartesian coordinates (in projections *x* and *y*), considering the relationship between cinematic and dynamic viscosity:

(5) $$\begin{array}{c} \frac{\partial u}{\partial t}+u\frac{\partial u}{\partial x}+\upsilon \frac{\partial u}{\partial y}=F_{x}-\frac{1}{\rho }\frac{\partial P}{\partial x}+\nu \cdot \nabla^{2}u\\ \frac{\partial \upsilon }{\partial t}+u\frac{\partial \upsilon }{\partial x}+\upsilon \frac{\partial \upsilon }{\partial y}=F_{y}-\frac{1}{\rho }\frac{\partial P}{\partial y}+\nu \cdot \nabla^{2}\upsilon \end{array}$$

where *u*, *υ*—projections of the velocity vector on *x* and *y* axes, (m/s); *F_x_*, *F_y_*—projections of the main vector of bulk forces on *x* and *y* axes, (N); *t*—time, (s); *ν*—kinematic viscosity of polymer melt, (St) is equal to the ratio of dynamic viscosity *μ,* and *P* is equal to constant density *ρ*, (kg/m^3^).

Let us write the condition of incompressibility of polymer melt in Cartesian coordinates (in projections x and y):

(6) $$\frac{\partial u}{\partial x}+\frac{\partial \upsilon }{\partial y}=0$$

The projections of the main vector of bulk forces can be determined by Newton’s second law:

(7) $$F_{x}=\rho \cdot S\cdot h\cdot \frac{\partial \upsilon_{x}}{\partial t},\;F_{y}=\rho \cdot S\cdot h\cdot \frac{\partial \upsilon_{y}}{\partial t}$$

where *S*—cross-section area of a mold cavity along its axis, (m^2^); *h*—height of a mold cavity along its axis, (m).

Due to the fact that at this stage, a plane problem is considered—i.e., the motion of the polymer melt occurs in the plane of the mold cavity—its height *h* and, accordingly, projections of the main vector of bulk forces *F_x_*, *F_y_*, are equal to zero. With this in mind and according to the incompressibility condition (6), the system of equations describing the motion of polymer melt in a mold cavity (5) will be written as follows:

(8) $$\begin{array}{c} \frac{\partial u}{\partial t}+u\frac{\partial u}{\partial x}+\upsilon \frac{\partial u}{\partial y}=-\frac{\partial p}{\partial x}+\nu \cdot \left[\frac{\partial^{2}u}{\partial x^{2}}+\frac{\partial^{2}u}{\partial y^{2}}\right]\\ \frac{\partial \upsilon }{\partial t}+u\frac{\partial \upsilon }{\partial x}+\upsilon \frac{\partial \upsilon }{\partial y}=-\frac{\partial p}{\partial y}+\nu \cdot \left[\frac{\partial^{2}\upsilon }{\partial x^{2}}+\frac{\partial^{2}\upsilon }{\partial y^{2}}\right] \end{array}$$

where *p*—the ratio of pressure *P* to constant density *ρ* (hereinafter simply “pressure”).

To determine pressure, we use the Poisson equation:

(9) $$\nabla^{2}p=\frac{\partial^{2}p}{\partial x^{2}}+\frac{\partial^{2}p}{\partial y^{2}}=-\frac{\partial D}{\partial t}-u\frac{\partial D}{\partial x}-\upsilon \frac{\partial D}{\partial y}+\nu \cdot \nabla^{2}D$$

where *D*—the divergence of the velocity vector, which is determined by the following relation:

(10) $$D=\frac{\partial u}{\partial x}+\frac{\partial \upsilon }{\partial y}$$

Therefore, the systems of Equations (8) and (9) are basic for determining velocity and pressure fields. If we take into account the assumption that the flow of viscous fluid is incompressible, i.e., take into account Condition (6), Equation (9) will become the Laplace equation for pressure *p*:

(11) $$\nabla^{2}p=0$$

**Figure 1:**
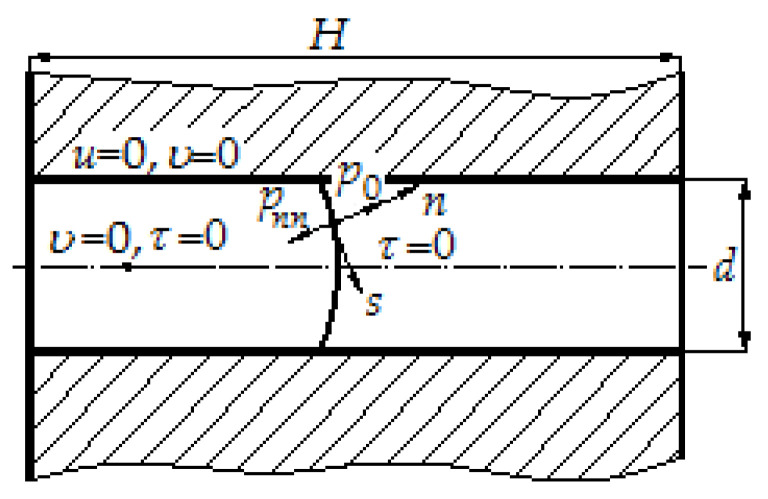
Boundary conditions for model of filling mold cavity with polymer melt.

We add boundary conditions on the axis of symmetry, at the entrance to the mold cavity, on solid surfaces of the mold cavity, on the free surface of polymer melt (Figure 1).

1. Boundary conditions on the axis of symmetry. According to [25,27], we assume that on the axis of symmetry, the radial velocity *υ* and tangential stress *τ* are equal to zero.
2. Boundary conditions near solid surfaces. According to [27], we assume that at the points where the polymer melt touches solid walls, its velocity goes to zero, i.e., at the points of contact of polymer melt with solid walls, its normal and the tangent velocity components are equal to zero: *u* = 0, *υ* = 0. The boundary conditions for pressure near solid walls and on the axis of symmetry are unknown, they can be found from the system of Equation (8) by substituting the limiting velocities in them.
3. Boundary conditions at the entrance to the mold cavity. According to [25], one of the components of the velocity vector is given here. The initial conditions of the problem are the values of velocities and pressure at the initial time.
4. Boundary conditions on the free surface. On the free surface, where polymer melt is assumed to flow out of the region under consideration, so-called "soft" boundary conditions are applied. Here, we assume the equality of normal pressure derivatives and velocity components to zero:
  (12) $$\begin{array}{c} \frac{\partial p_{n}}{\partial x}=0\\ \frac{\partial u}{\partial x}=0\\ \frac{\partial \upsilon }{\partial x}=0 \end{array}$$

In this paper, we consider relatively slow motions of viscous liquids, and according to the theory of viscous medium [22] and taking into account the slow motion of the dense polymer melt, Equation (8) can be written as follows:

(13) $$\begin{array}{c} \frac{\partial p}{\partial x}=\nu \cdot \left[\frac{\partial^{2}u}{\partial x^{2}}+\frac{\partial^{2}u}{\partial y^{2}}\right]-\frac{\partial u}{\partial t}\\ \frac{\partial p}{\partial y}=\nu \cdot \left[\frac{\partial^{2}\upsilon }{\partial x^{2}}+\frac{\partial^{2}\upsilon }{\partial y^{2}}\right]-\frac{\partial \upsilon }{\partial t} \end{array}$$

If we use the hypothesis of the quasi-stationary character of flow [27], i.e., the whole process of motion of polymer melt is divided into a finite number of stationary problems, the system of Equation (13) can be represented as follows:

(14) $$\begin{array}{c} \frac{\partial p}{\partial x}=\nu \cdot \left[\frac{\partial^{2}u}{\partial x^{2}}+\frac{\partial^{2}u}{\partial y^{2}}\right]\\ \frac{\partial p}{\partial y}=\nu \cdot \left[\frac{\partial^{2}\upsilon }{\partial x^{2}}+\frac{\partial^{2}\upsilon }{\partial y^{2}}\right] \end{array}$$

We will solve Equation (11) and the system of Equation (14) with given boundary conditions using the finite-difference method of markers and cells [28]. The markers can move along the Euler grid with the local velocity of polymer, showing its position at a certain point in time according to the Lagrange equation:

(15) $$\begin{array}{c} \frac{dx}{dt}=u\\ \frac{dy}{dt}=\upsilon \end{array}$$

**Figure 2:**
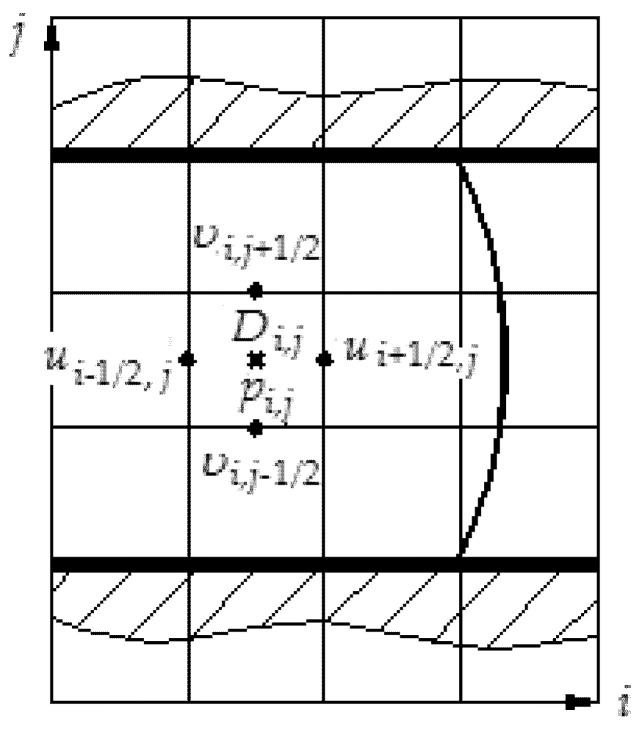
Calculation scheme.

In this way, markers are introduced only to visualize the flow of polymer melt. They show which cells contain polymer material (filled cells) and which do not contain it (empty cells). Their main task is to visualize the flow of polymer melt. A typical cell is shown in Figure 2.

In order for polymer melt to flow through this cell, the condition of incompressibility must be fulfilled, i.e., the distance between the markers located within a unit cell must not change during the flow.

In each cell, three independent variables *u*, *υ,* and *p* are defined, and each of them is centered at separate points on the grid. Pressure *p* and divergence *D* are given in the center *i*, *j* of each cell.

The horizontal velocity component $u_{i+1/2,j}$ is determined only at the vertical cell boundaries $\left(x_{i+\frac{1}{2}},y_{j}\right)$ and, similarly, the vertical velocity component $\upsilon_{i,j+1/2}$ is determined only at the horizontal cell boundaries $x_{i},y_{j+1/2}$.

In the future, to avoid half-integer indices $u_{i+1/2,j}$ and $\upsilon_{i,j+1/2}$, we will denote $u_{i,j}$ and $\upsilon_{i,j}$ velocities with indexation referring to the center of the cell, remembering that they are centered in the middle of the sides of the cell.

As noted earlier, boundary conditions near solid walls contain normal pressure derivatives. To satisfy these conditions, it is necessary to introduce fictitious cells that are located outside the physical solid boundary [29].

Markers moving at local velocities are distributed along the Euler grid. As a result, the configuration of viscous fluid is determined by the distribution of markers on the Euler grid, which consists of four types of cells [27]:

- Boundary cells, in which the conditions are met near solid surfaces of the mold cavity, at the entrance to the mold cavity, on the axis of symmetry;
- Full cells that contain markers and are surrounded by other full cells;
- Empty cells that do not contain markers; and
- Surface cells that contain markers but border on empty cells.

Using [22,25], we will write the differential equations of Motion (14), which describe the flow of polymer melt, in finite-difference form, replacing the differential operators with their difference analogues:

(16) $$\frac{\left(p_{i,j}-p_{i+1,j}\right)}{h}=\left(\frac{\nu }{h^{2}}\right)\cdot \left(u_{i+1,j}+u_{i-1,j}+u_{i,j+1}+u_{i,j-1}-4u_{i,j}\right)=0$$

(17) $$\frac{\left(p_{i,j}-p_{i,j+1}\right)}{h}=\left(\frac{\nu }{h^{2}}\right)\cdot \left(\upsilon_{i+1,j}+\upsilon_{i-1,j}+\upsilon_{i,j+1}+\upsilon_{i,j-1}-4\upsilon_{i,j}\right)=0$$

The finite difference analogue of the continuity Equation (6) is written in the form:

(18) $$D_{i,j}=\frac{\left(u_{i,j}-u_{i-1,j}+\upsilon_{i,j}-\upsilon_{i,j-1}\right)}{h}=0$$

The pressure field according to Equation (11) in difference form will be written as:

(19) $$\frac{\left(p_{i+1,j}+p_{i-1,j}+p_{i,j+1}+p_{i,j-1}-4p_{i,j}\right)}{h^{2}}=0$$

Similarly, in the finite differences, we will write boundary conditions for cells near the solid surface of the mold cavity, at the entrance to the mold cavity, on the axis of symmetry, and on the free surface of the melt [19].

The above expressions form a complete system of linear algebraic equations to determine all the unknowns in different types of cells: boundary, full, and surface ones. To solve these systems of equations, it is advisable to use iterative methods that take into account the special form of such systems and are convenient for implementation on a computer [26].

The iterative Liebman method was used in the work [30], with consecutive relaxations:

(20) $$\begin{array}{c} \frac{dx}{dt}=u\\ \frac{dy}{dt}=\upsilon \end{array}$$

where *ω*—the relaxation parameter that takes values from 0 to 2. At *ω* > 1, the process is called “upper” relaxation; at *ω* < 1, the process is called “lower” relaxation.

The modeling results of the processes that occur during the structure formation of material with filler particles make it possible to design and engineer molding equipment for the production of light industry polymer products with high performance properties.

### 3.2. Simulation of Mold Cavity Filling Process with Polymer Melt-Containing Inclusions

**Figure 3:**
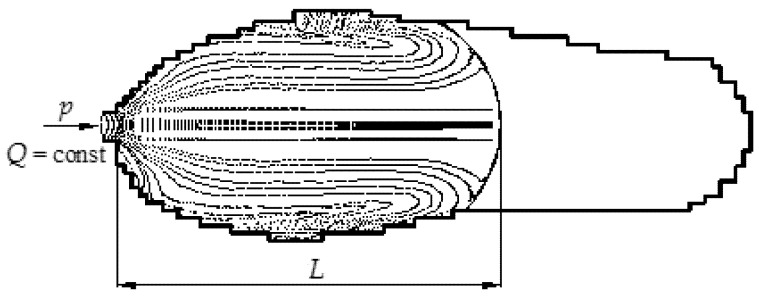
Simulation of filling mold cavity for making soles with polymer melt.

When polymer melt is injected through the inlet channel into the mold cavity, it begins to flow in the radial direction [30]. As the perimeter of the flow front increases, the melt is subjected to elastic stretching at the right angle to the flow direction. While the mold cavity is filled with melt, a flow velocity profile is set in the direction of its width (Figure 3). The highest velocity of the polymer melt is in the middle of the flow, and at the edges, it decreases to zero due to adhesion to the cold walls of the mold cavity. As a result, the melt is subjected to shear forces, which create resistance to the flow, determining the molding pressure.

However, the shear of material is not uniform in the direction of width of the mold cavity. Moreover, near the solid walls, there is a layer with significant shear, while in the center, the melt flows faster and almost without shear. Part of the melt that flows very rapidly forward in the middle layer is eventually directed by a “fountain-like” front flow in close proximity to the solid walls of the mold cavity and thus is affected by shear stresses. The initial orientation of the filler particles does not affect their final orientation.

**Figure 4:**
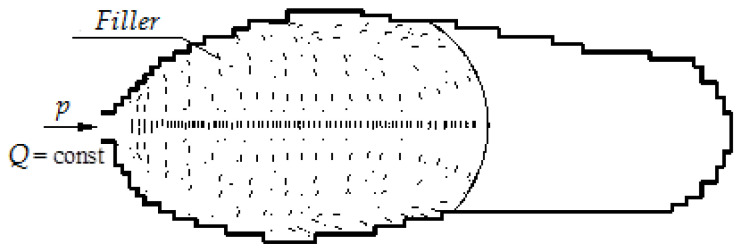
Simulation of filling mold cavity with polymer melt-containing filler.

Thus, the orientation of inclusions is formed as a result of stretching, the “fountain-like” front flow of polymer melt and under the influence of shear stresses (Figure 4).

As can be seen from Figure 4, the processes of filler particles orientation in the mold cavity overlap. Melt together with inclusions, entering the cavity through the inlet channel, at the beginning receives a predominant transverse orientation; then, it moves with the flow in the center of the cavity in the direction of the melt front without any change in orientation, i.e., inclusions are still oriented across the flow direction [31]. At the front, the melt with inclusions is redirected to the walls of the mold, and as a result, it enters the shear flow layer, which reorients the inclusions. Thus, as a result of both processes, two layers appear in the polymer product, in each of which the main direction of the particles is rotated by 90° relative to the other one.

The mechanical properties of the products will depend on the thickness of these layers and the corresponding orientation of inclusions. In those places where the peripheral layers predominate, the orientation of the inclusions is parallel to the direction of the melt flow. Closer to the center of the flow, the orientation of inclusions is perpendicular to the direction of the flow. Thus, near the walls of the mold, a layer of polymer is formed with filler particles oriented along the flow of polymer melt, which prevents shrinkage of the products.

### 3.3. Design of Mold and Device for Feeding Particles of Recycled Waste

#### 3.3.1. Mold for Making Soles Using Mixture of Polymer Melt and Recycled Waste

**Figure 5:**
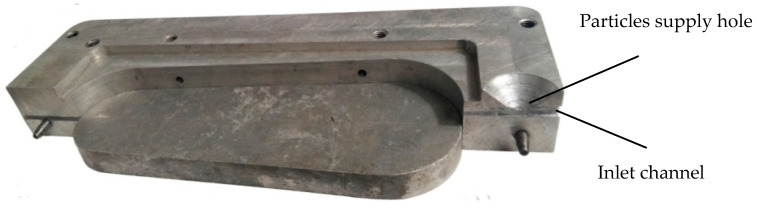
Mold for making soles with recycled polymer waste particles.

**Figure 6:**
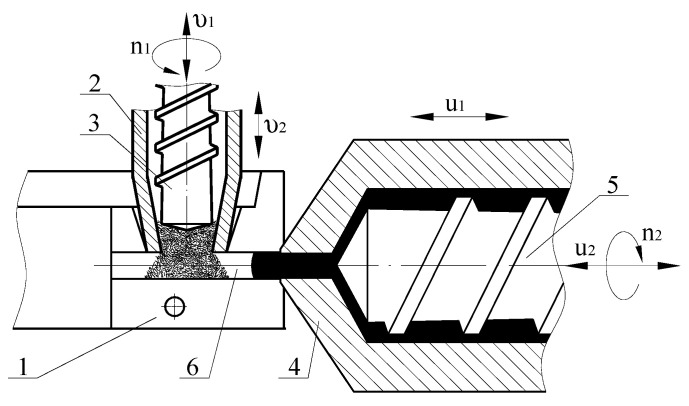
Design of device for feeding particles of recycled waste into inlet channel of mold: 1—mold; 2, 4—material cylinders of devices for feeding, respectively, particles of recycled waste and polymer melt; 3, 5—screws; 6—inlet channel.

Using the above model of the movement of polymer melt in the inlet channel and cavity of the mold for making shoe parts and taking into account the results obtained in [32], a method of designing molds for the injection molding of reinforced polymer products [30] was developed. A mold was designed [33,34] and manufactured, which consists of two semi-matrices (Figure 5) and a plate (Figure 6). The design of the mold allows making soles for footwear with inclusions of processed polymer waste.

Particles of recycled polymer waste were fed directly into the inlet channel of the mold using a specially designed device (Figure 6). As established in the previous section, the initial orientation of polymer particles does not affect their orientation in the finished product.

Screws 3 and 5, rotating, move in the directions away from mold 1. As a result, particles of recycled waste enter the inlet channel 6 of mold 1, and a homogeneous melt of polymer fills the dosing area of material cylinder 4 in front of screw 5. After accumulation of the required volume of waste particles and polymer melt, screws 3 and 5 stop and begin to move without rotation in the directions toward mold 1 with velocities that ensure a uniform distribution of particles in the volume of the sole.

#### 3.3.2. Parameters of Device for Feeding Recycled Waste Particles into Inlet Channel of Mold

The screw conveyor is the most convenient and easy to operate for moving polymer waste (Figure 6). This device transports processed polymer particles to the inlet channel of the mold.

At present, processes associated with the motion of molten polymer in a material cylinder are not sufficiently studied. In most works, the process of injection molding, filling of a mold cavity, and forming of products are studied [22,25,35]. At the same time, rather insignificant attention is paid to the movement of polymer material without its homogenization.

The transportation of well-flowing loose polymeric materials with a sufficiently large bulk density is not a serious problem. However, the transportation of polymer particles of different shapes and sizes (crushed waste) is quite a difficult task.

After particles of polymer material enter a screw channel, the movement of material becomes forced. Gravity forces may still play a minor role, but pushing forces predominate.

**Figure 7:**
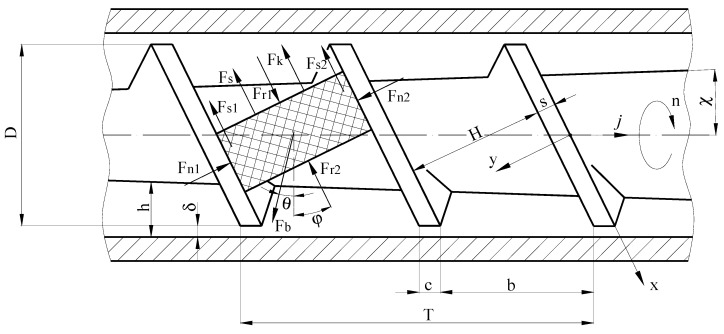
Scheme of forces acting on polymer particles.

The following forces act on the transported material (Figure 7): *F_b_*—friction force acting on the polymer particles on the side of the cylinder wall and causing it to move along the screw channel, (N); *F*_*r*1_ and *F*_*r*2_—reactions of compressed particles, (N); *F*_*n*1_ and *F*_*n*2_—normal forces acting on transported material on the side of the screw walls, (N); *F_s_*—the normal force acting on the polymer particles on the side of the screw core if this core is conical, (N); *F*_*s*1_ and *F*_*s*2_—friction forces, respectively, on the side of the pushing and frontal walls of the screw, (N); *F_k_*—friction force acting on particles on the side of the channel bottom, (N).

For the first time, a complete analysis of the movement of solid particles in a traditional single-screw extruder was performed in [36]. The analysis presented in this paper is extended to take into account all the above-mentioned forces [37,38].

When modeling the movement of polymer particles in a material cylinder, the basic assumption is that solid particles, when compacted, form conglomerates in which there are no shear deformations, and the compression of particles is viscoelastic. Particle conglomerates are subjected to frictional forces between them and the surfaces of the screw and the material cylinder [39]. To this assumption, we add the following: conglomerates of particles move in a continuous stream; a solid layer of particles is in contact with the entire surface of the channel; we neglect the gap over the screw ridge, gravitational forces, and the curvature of the channel.

**Figure 8:**
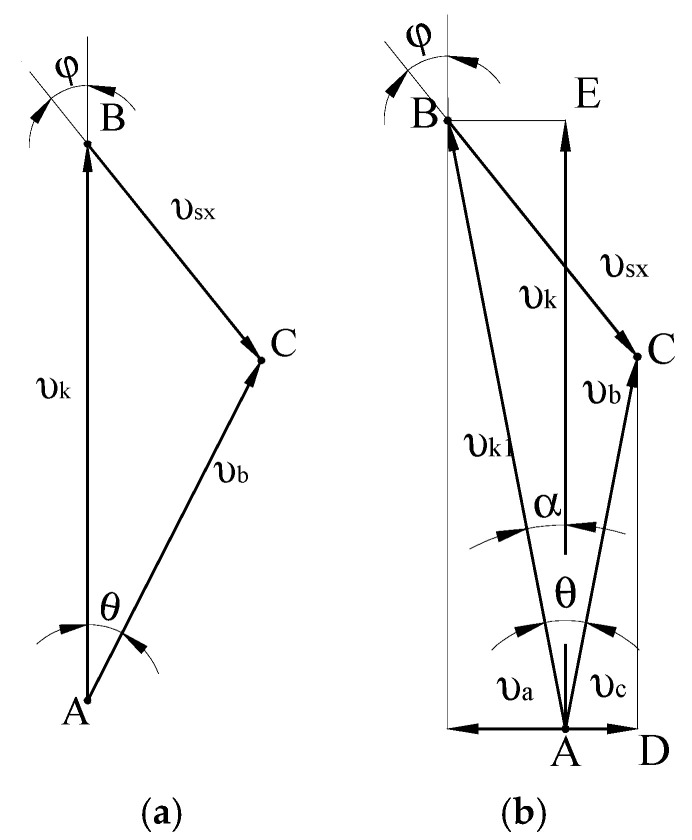
Velocity plan: (**a**) screw only rotates; (**b**) screw rotates and moves along axis.

Consider the mechanism of movement of grouped particles of recycled polymer waste in the helical channel of the screw, which rotates inside a material cylinder (Figure 8).

Choose point A (some point of the conglomerate of particles), which at the initial time coincides with the corresponding point of the helical channel. Assume that the screw rotates clockwise when viewed from the end of the material cylinder. Then, the point of the screw channel will move from the bottom to the top with the velocity *υ_k_* and in time *t_ab_* will move to a distance *AB*. Simultaneously, under the action of friction between particles of material and the inner wall of the material cylinder, the particles move from point *A* to point *C* with the velocity *υ_b_*.

Accordingly, along the channel, the particles will move down at a distance *BC* with the velocity *υ_sx_* (*x* is the axis directed along the helical channel development), which is determined by the angle the ridge helix φ. Thus, the movement angle of the conglomerates of recycled waste particles *θ* is the angle between the velocity of the screw and the velocity of the polymer *υ_b_* relative to the wall of the material cylinder.

In the case when the screw in addition to rotation has an axial movement, the velocity plan is constructed as follows (Figure 8b). As can be seen from Figure 8b, point *A* moved to point *B* in time *t_ab_*, but it moved with the velocity *υ*_*k*1_, which consists of tangential (circular) *υ_k_* and axial (along the axis of the screw) *υ_a_* velocities. The angle *α* between *υ_k_* and *υ*_*k*1_ is determined from the following equation

(21) $$\mathrm{tg}\alpha =\frac{\upsilon_{a}}{\upsilon_{k}}$$

where $\upsilon_{k}=\pi Dn$—the angular velocity of the screw (rad/s); *n*—the circular speed of the screw, (s^−1^); *D*—the diameter of the screw, (m).

It is obvious that the velocity *υ_sx_* of polymer particles moving along the channel is not affected by the velocity of the axial movement of the screw, so it can be determined from the triangle *ABC* (Figure 8a):

(22) $$\upsilon_{sx}=\upsilon_{k}\cdot \frac{\sin\theta }{\sin\left(\theta +\phi \right)}=\pi Dn\cdot \frac{\sin\theta }{\sin\left(\theta +\phi \right)}$$

In the case of a single-path screw, i.e., *i* = 1, the volumetric velocity of material can be determined from the following equation:

(23) $$Q=\upsilon_{sx}H\cdot h$$

where *H*—the width of the screw channel, (m); *h*—the depth of the helical channel, (m).

Substituting in Equation (23) the formula for determining the velocity $\upsilon_{sx}$ and the expression for determining the width of the channel presented in [37], we obtain:

(24) $$Q=\pi Dn\cdot \frac{\sin\theta }{\sin\left(\theta +\phi \right)}\cdot \pi Dh\cdot \sin\phi =\pi^{2}D^{2}hn\cdot \frac{\sin\theta \cdot \sin\phi }{\sin\left(\theta +\phi \right)}$$

The angle of movement of material *θ* is determined by considering steady motion and assuming that the friction forces acting on conglomerates of particles on the part of the helical channel (*F*_*s*1_, *F*_*s*2_, *F_k_*) and the friction forces acting on the part of the inner surface of the material cylinder (*F_b_*) can be related by the following formula [36]:

(25) $$F_{\Sigma }=F_{s1}+F_{s}+F_{k}=F_{b}\cos(\theta +\phi )$$

Assuming that the reaction of the walls of the screw channel and the material cylinder is the same, to determine the angle of movement *θ*, the following equation can be used [37]:

(26) $$\theta =\arcsin\left(\frac{\sqrt{1+f_{s}^{2}-k^{2}}}{1+f_{s}^{2}}\right)-\phi$$

where

(27) $$k=\frac{H}{i\cdot f_{s}}\ln\frac{P}{P_{0}}+\frac{f_{s}}{f_{b}}\left(1+\frac{2h}{H}\right)$$

Here, *P*—pressure field along the helical channel of screw, (Pa); *P*_0_—pressure at the beginning of the helical channel, (Pa); *f_b_*, *f_s_*—coefficients of friction between polymer particles and, accordingly, the inner wall of the material cylinder and walls of the screw channel.

The expression for determining the pressure field along the screw channel is obtained on the basis of the equation of balance of forces in the longitudinal and transverse directions of the channel. Before constructing the force balance equation, we determine all the forces acting on the particle conglomerate element during its movement along the screw channel.

The force of friction acting on the material on the side of the wall of the material cylinder that causes it to move along the helical channel of the screw can be determined from the following equation:

(28) $$F_{b}=f_{b}P_{b}Hdx=f_{b}K_{s}PHdx$$

where *P_b_*—pressure of polymer on the wall the material cylinder, (Pa); *P*—specific pressure of the conglomerate of recycled waste particles, (Pa); *K_s_* = *P_b_*/*P*—coefficient characterizing anisotropy of the pressure field.

The coefficient *K_s_* can also be determined from the following equation [36]:

(29) $$K_{s}=\frac{\left[1-\sin(\mathrm{arctg}f)\right]}{\left[1+\sin(\mathrm{arctg}f)\right]}$$

where *f*—coefficient of friction between the particles of the polymer material.

The axial and tangential components $F_{b}$, respectively, can be defined as follows:

(30) $$\begin{array}{c} F_{bj}=f_{b}P_{b}H\cdot \sin\theta \cdot dx=f_{b}K_{s}PH\cdot \sin\theta \cdot dx\\ F_{b\theta }=f_{b}P_{b}H\cdot \cos\theta \cdot dx=f_{b}K_{s}PH\cdot \cos\theta \cdot dx \end{array}$$

The reactions of compressed particles can be determined by multiplying the specific pressure by the cross-sectional area of the mold cavity, i.e.,

(31) $$F_{r1}=H\cdot h\cdot P,\;F_{r2}=H\cdot \left(h-dx\cdot \mathrm{tg}\chi \right)\cdot \left(P+dP\right)$$

If the screw core is conical, then the depth of the helical channel gradually decreases along its length, i.e., we will have a resultant force *F_r_* equal to the difference between the reactions of compressed particles:

(32) $$F_{r}=F_{r2}-F_{r1}=H\cdot \left(h-dx\cdot \mathrm{tg}\chi \right)\cdot \left(P+dP\right)-H\cdot h\cdot P=dP\cdot H\left(h-dx\cdot \mathrm{tg}\chi \right)-P\cdot H\cdot dx\cdot \mathrm{tg}\chi$$

The normal forces acting on the side of the walls of the screw channel on the particles can be determined as follows:

(33) $$\begin{array}{c} F_{n2}=P_{n}hdx=PK_{s}hdx\\ F_{n1}=PK_{s}hdx \end{array}$$

where *F*_*n*1_—the normal force acting on the side of the front wall of the screw channel, (N); *F*_*n*2_—the normal force acting on the side of the pushing wall of the screw channel, N.

In screws with a conical core, there is the additional normal force acting on the side of the core, which is determined by the following equation [36]:

(34) $$F_{s}=PK_{s}H\chi dx$$

The friction forces acting on the material on the side of the pushing and front walls of the screw channel, respectively, are equal to:

(35) $$\begin{array}{c} F_{s2}=F_{n2}\cdot f_{s}=PK_{s}hf_{s}dx\\ F_{s1}=F_{n1}\cdot f_{s}=\left(PK_{s}hdx+F^{*}\right)f_{s} \end{array}$$

The friction force acting on material on the side of the channel bottom is equal to:

(36) $$F_{k}=PK_{s}Hf_{s}dx$$

During normal operation of the screw conveyor, the sum of the projections of all forces on the screw axis must be zero, i.e.,

(37) $${\left(F_{b}\right)}_{j}+{\left(F_{r}\right)}_{j}+{\left(F_{s}\right)}_{j}+{\left(F_{n1}-F_{n2}\right)}_{j}+{\left(F_{s1}-F_{s2}\right)}_{j}+{\left(F_{k}\right)}_{j}=0$$

**Figure 9:**
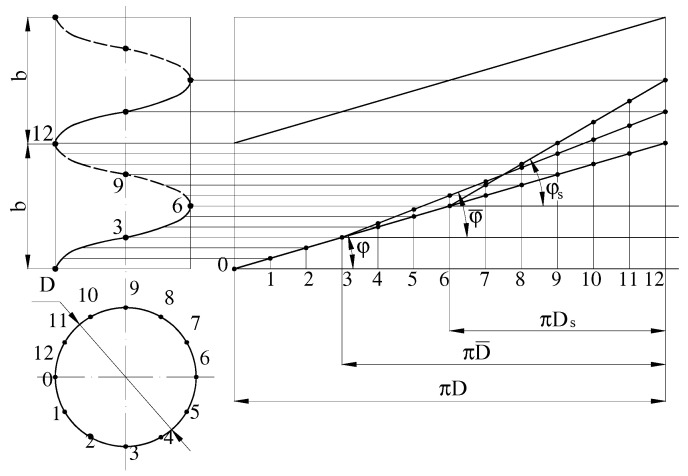
Screw development.

We obtain the axial components of the forces by multiplying them by sin φ, $\sin\bar{\varphi }$, and $\sin\varphi_{s}$ (Figure 9).

The forces acting in the plane of the channel bottom are multiplied by $\sin\varphi_{s}$; the forces acting in the planes of the side surfaces of the channel are multiplied by $\sin\bar{\varphi }$; the forces acting in the plane of contact of material and the material cylinder wall are multiplied by sin φ:

(38) $$F_{b}\cdot \sin\phi +F_{r}\cdot \sin\bar{\phi }+F_{s}\cdot \sin\phi_{s}+\left(F_{n1}-F_{n2}\right)\cdot \sin\bar{\phi }+\left(F_{s1}-F_{s2}\right)\cdot \sin\bar{\phi }+F_{k}\cdot \sin\phi_{s}=0$$

We write the equation of equilibrium in the moments relative to the screw axis. To do this, each force is multiplied by the corresponding radius value. If the forces are applied in the plane of the screw channel bottom, the diameter is equal to $D_{s}=D-2h$; if the forces are applied in the planes of the channel side surfaces, the diameter is equal to $\bar{D}=D-h$. Taking into account the above equation of equilibrium in the moments relative to the screw axis will be written as follows:

(39) $${\left(F_{b}\right)}_{\theta }\cdot \frac{D}{2}-{\left(F_{r}\right)}_{\theta }\cdot \frac{\bar{D}}{2}-{\left(F_{s}\right)}_{\theta }\cdot \frac{D_{s}}{2}-{\left(F_{n1}-F_{n2}\right)}_{\theta }\cdot \frac{\bar{D}}{2}-{\left(F_{s1}-F_{s2}\right)}_{\theta }\cdot \frac{\bar{D}}{2}-{\left(F_{k}\right)}_{\theta }\cdot \frac{D_{s}}{2}=0$$

Similarly, we obtain tangential components of forces by multiplying the modulus of the corresponding force vector by cos φ, $\cos\bar{\varphi }$, and $\cos\varphi_{s}$, depending on the plane of the force action (Figure 9).

(40) $$F_{b}\cdot \cos\phi \cdot \frac{D}{2}-F_{r}\cdot \cos\bar{\phi }\cdot \frac{\bar{D}}{2}-F_{s}\cdot \cos\phi_{s}\cdot \frac{D_{s}}{2}-\left(F_{n1}-F_{n2}\right)\cdot \cos\bar{\phi }\cdot \frac{\bar{D}}{2}-\left(F_{s1}-F_{s2}\right)\cdot \cos\bar{\phi }\cdot \frac{\bar{D}}{2}-F_{k}\cdot \cos\phi_{s}\cdot \frac{D_{s}}{2}=0$$

Converting Equations (38) and (40) and performing integration within the distance of transportation of polymer waste, we obtain the expression for determining inlet pressure:

(41) $$P_{2}=P_{1}\cdot e^{\left(\int_{z_{1}}^{z_{2}}\frac{B_{1}-A_{1}K}{A_{2}K+B_{2}}dz\right)}$$

where *P*_1_—pressure at beginning of polymer waste transportation, i.e., at a distance *z*_1_ = 0, (Pa); *P*_2_—pressure at end of transportation, i.e., at a distance *z*_2_, (Pa).

(42) $$\begin{array}{c} A_{1}=Hf_{b}K_{b}\sin\theta -H\chi \sin\bar{\phi }+2hK_{s}f_{s}\sin\phi +Hf_{s}K_{s}\frac{\sin\phi \left(1+\frac{\chi }{f_{s}}\right)}{\sqrt[{}]{1+\chi^{2}}}\\ B_{1}=Hf_{b}K_{b}\cos\theta +H\chi \cos\bar{\phi }\frac{\bar{D}}{D}-2hK_{s}f_{s}\sin\phi \cdot \mathrm{ctg}\bar{\phi }\frac{\bar{D}}{D}-Hf_{s}K_{s}\left(1+\frac{\chi }{f_{s}}\right)\frac{D_{s}\mathrm{ctg}\phi_{s}}{D\sqrt[{}]{1+\chi^{2}}}\\ B_{2}=\frac{Hh\bar{D}\cos\bar{\phi }}{D}\\ K=\frac{\bar{D}}{D}\frac{\sin\bar{\phi }+f_{s}\cos\bar{\phi }}{\cos\bar{\phi }-f_{s}\sin\bar{\phi }}\\ \bar{\phi }=\mathrm{arctg}\left(\frac{b}{\pi \bar{D}}\right)\\ \phi_{s}=\mathrm{arctg}\left(\frac{b}{\pi D_{s}}\right) \end{array}$$

Equation (41) is solved by a numerical method. To simplify the model, we replace a conical core with a stepped cylindrical one, i.e., substitute *χ* = 0 in the equations. Assuming that the conglomerates of recycled waste particles are isotropic medium, i.e., $K_{s}=K_{b}=1$, we obtain the following expressions:

(43) $$P_{i+1}=P_{i}\cdot e^{\frac{B_{1}-A_{1}K}{A_{2}K+B_{2}}\Delta z}$$

where

(44) $$\begin{array}{c} A_{1}=Hf_{b}\sin\theta +2hf_{s}\sin\phi +Hf_{s}\sin\phi \\ B_{1}=Hf_{b}\cos\theta -2hf_{s}\sin\phi \cdot \frac{\bar{D}}{D}\mathrm{ctg}\bar{\phi }-Hf_{s}\frac{D_{s}}{D}\mathrm{ctg}\phi_{s}\\ B_{2}=\frac{Hh\bar{D}\cos\bar{\phi }}{D}\\ K=\frac{\bar{D}}{D}\cdot \frac{\sin\bar{\phi }+f_{s}\cos\bar{\phi }}{\cos\bar{\phi }-f_{s}\sin\bar{\phi }} \end{array}$$

**Figure 10:**
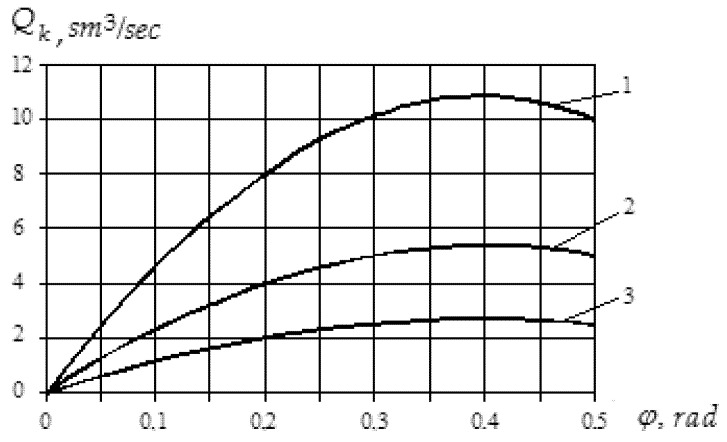
Graphs of dependence of volumetric velocity of particles on angle of elevation of helical channel: 1—*n* = 100 min^−1^; 2—*n* = 50 min^−1^; 3—*n* = 25 min^−1^.

**Figure 11:**
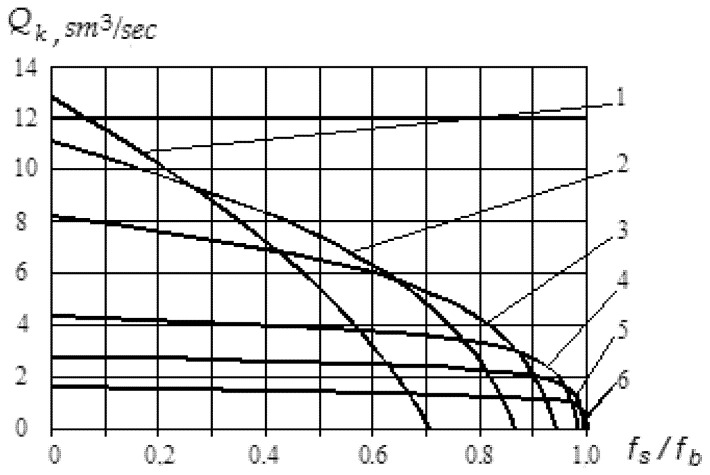
Graphs of dependence of volumetric velocity of particles on ratio coefficients of friction: 1—angle of elevation of helical channel φ = 45°; 2—φ = 30°; 3—φ = 20°; 4—φ = 10°; 5—φ = 6°; 6—φ = 3°.

As a result of theoretical research using data from [36,40], it was found that the influence of the ratio of friction coefficients between polymer and the cylinder and between polymer and the screw on the volumetric velocity of material is significant (Figure 10 and Figure 11). The obtained results are in good agreement with the results of the experimental studies presented in [40].

Thus, the movement of polymer particles at a large angle of elevation of the helical channel requires a more specific ratio of coefficients of friction than for small angles (Figure 10). For example, if the elevation angle is close to 90°, then a stable process of transporting the particles of recycled waste into the inlet channel of the mold can be provided only if the coefficient of friction between the screw and polymer material is close to zero. It is clear that such conditions are not real. On the other hand, as the angle of elevation of the helical channel decreases, larger values of the ratio of the coefficients of friction become acceptable (Figure 11).

For example, a screw with an angle of elevation of the helical channel *ϕ* = 10° at a ratio of friction coefficients *f_s_/f_b_* = 0.2 will provide a twice as low volumetric velocity than a screw with an angle of elevation *ϕ* = 60°, and a ratio of friction coefficients *f_s_*/*f_b_* = 0.65 will provide twice greater the volumetric velocity.

The analysis showed that at small angles of elevation of the helical channel, the best conditions for the transportation of processed particles of polymer material are created, and this angle should lie in the range between 19° (0.33 rad) and 24° (0.42 rad). The obtained results are in good agreement with those of [36,40].

Therefore, the above analytical dependences can be used to determine the rational design parameters of the device for transporting polymer particles and effective modes of feeding them into the inlet channel of the mold, which provides the necessary distribution of particles in the volume of the sole. For example, you can change the volumetric velocity of polymer particles in the inlet channel of the mold by changing the roughness of the surfaces of the screw and the inner walls of the material cylinder (the use of antifriction coating, the use of catalytic surface conversion: J-Tex or Dyna-blue); friction area (add certain elements or cut grooves); the temperature of the material cylinder.

**Figure 12:**
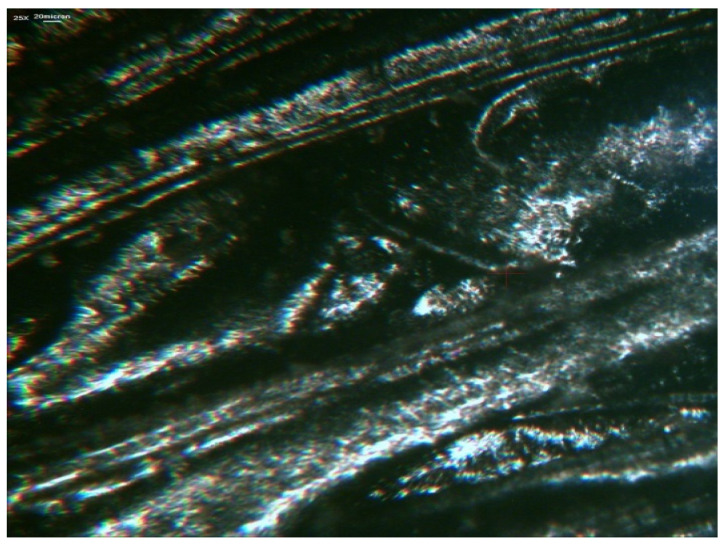
Photomicrograph of longitudinal section of polyvinylchloride sole made with addition of waste particles.

The use of a scanning electron microscope allowed presenting the structure of polymer obtained by injection molding with the addition of polymer particles (Figure 12).

A common and important characteristic for almost all types of filled polymer products is the length of the particles of recycled polymer waste, which is determined by the design and technological parameters of processing equipment.

The main correct method for evaluating the physical and mechanical properties of filled polymers can be to determine the tensile strength. In the process of stretching the filled polymer products, the particles endure tensile load and the main polymer (matrix): a shear one. Therefore, the polymer surrounding the particles must withstand a shear load equal to the tensile load of the particle.

The strength of the polymer is much lower than the strength of the particle with an oriented structure. To ensure high strength of the filled polymer products according to [41], the ratio of the particle length to its transverse size $\frac{l}{d}$ must be at least 10.

**Figure 13:**
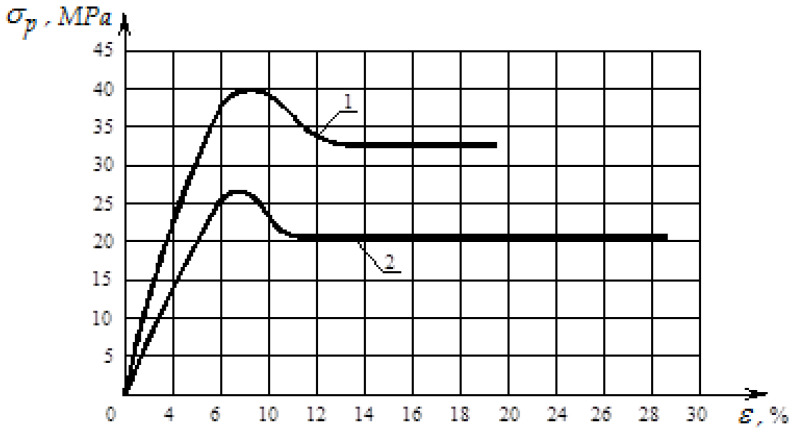
Stress–strain curve for polymer samples filled with long particles of polyvinylchloride: 1—along orientation of particles; 2—across orientation of particles.

**Figure 14:**
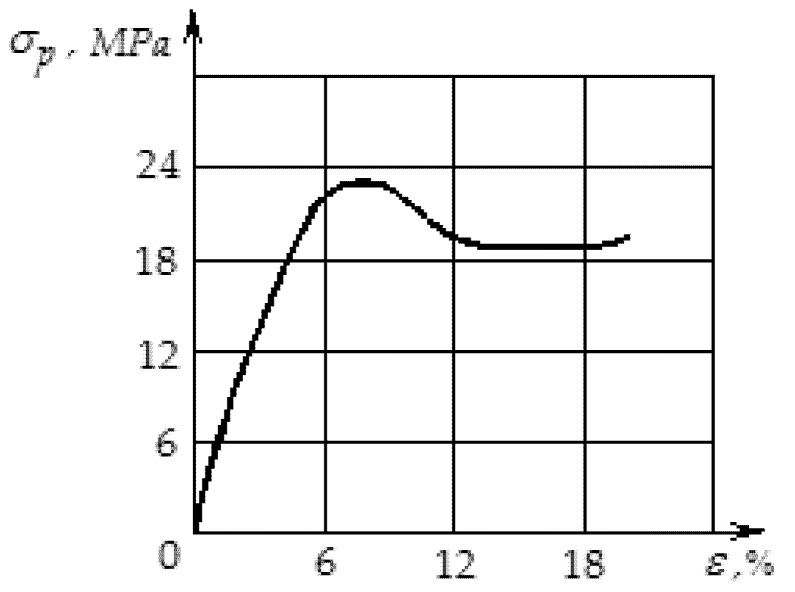
Stress–strain curve for polymer samples filled with short particles of polyvinylchloride.

In order to determine the effect of particle sizes of recycled polyvinylchloride waste on the physical and mechanical properties of soles for shoes made of polyvinylchloride, experimental studies of the relationships between tensile stresses and deformations of shoe soles at different particle sizes were conducted. The studies were conducted for polyvinylchloride soles with the inclusion of short ($\frac{l}{d}=4\ldots 6$) and long ($\frac{l}{d}=4\ldots 6$) particles of recycled polyvinylchloride. The transverse particle size was about 1 mm (Figure 13 and Figure 14).

Figure 13 shows the graph of the dependence of tensile stress on the deformation of polymer samples cut from the filled sole along and across the orientation of the particles of polyvinylchloride of a long fraction.

Figure 14 shows the graph of the dependence of tensile stress on the deformation of polymer samples cut from the filled sole along the orientation of the polyvinylchloride particles of a short fraction.

## 4. Discussion

The experimental tests of the breaking strength and fatigue life of the soles made by injection molding from a mixture of PVC and particles of recycled polyvinyl chloride have shown that the concentration and orientation of the recycled polymer waste particles in the finished product substantially affect its performance.

As can be seen from Figure 13, the influence of particle length on the properties of the sole is significant. Thus, improving the performance of polymer products of light industry can be provided by filling them with long particles, which must be oriented along the direction of action of loads during the operation of these products. To ensure a greater flexibility of the polymer product without its rupture, long particles should be oriented across that direction.

From Figure 14, it follows that the shorter the filler particles, the lower their efficiency. With short particles, the matrix under no circumstances can transmit to the particles a stress sufficient to destroy them.

Thus, the reinforcing capacity of short particles (increasing the performance properties of the polymer product) is quite low, taking into account the orientation of the particles, which at injection molding or the extrusion of polymer products is not ideal.

The shorter the filler particles, the less efficient they are. In the case of short particles, the matrix cannot provide the particles with tensile stress sufficient to destroy them. Thus, the reinforcing ability of short particles (increasing the operational properties of a polymeric product) is rather low, especially taking into account the particle orientation, which is not ideal for molding or extruding polymer products.

It has also been established that for 10% concentration of the recycled polyvinylchloride waste particles in the process of injection molding, their durability and fatigue endurance when loaded along the orientation of the particles increased by 28% and 14%, respectively, and across the orientation, they increased by 9% and 12%. With an increase in the recycled waste concentration by more than 15%, the strength and endurance of the sole is reduced.

## 5. Conclusions

New technological processes and equipment for the injection molding of polymer products of light industry filled with recycled polymer waste with increased performance characteristics have been developed. The manufacturing of such products was accomplished on the basis of the development of a mathematical model for movement of the mixture of polymer material and particles of recycled polymer waste in the process of filling the mold cavity, which, in contrast to the existing models, allows observing formation of the polymer product structure containing recycled waste particles.

On the basis of the obtained results, a mold for experimental studies of the influence of its design parameters and technological characteristics of injection molding on the formation of the shoe sole structure with reinforcing particles of polymer waste was designed and manufactured. The process of the mold filling and the shoe sole structure forming from a mixture of polyvinyl chloride and particles of recycled waste from polyvinyl chloride was conducted.

Analytical dependencies that connect the parameters of the device for transporting recycled polymer waste particles to the molding inlet channel with the particle bulk feed rate have been developed to determine rational structural parameters of the device and efficient transport modes for which a necessary concentration and distribution of particles in the sole volume is achieved.

Improvement in the performance characteristics of shoe soles made by the injection molding of a mixture of polyvinylchloride and particles of recycled polyvinylchloride was confirmed by experimental tests of breaking strength and fatigue life. The results of these tests can be used in the design of processing equipment to obtain particles of the required shape and size and in the design of molds to provide the required concentration and orientation of particles in polymer products of light industry.

Taking into account the obtained results, future research is needed for the development of processing equipment for manufacturing various types of products from polymer materials with the addition of reinforcing particles of recycled polymer waste.
